# Clinical Features and Experimental Models of Cerebral Small Vessel Disease

**DOI:** 10.3389/fnagi.2020.00109

**Published:** 2020-05-05

**Authors:** Akihiro Shindo, Hidehiro Ishikawa, Yuichiro Ii, Atsushi Niwa, Hidekazu Tomimoto

**Affiliations:** Department of Neurology, Mie University Graduate School of Medicine, Mie University, Tsu, Japan

**Keywords:** white matter, lacuna, Alzheimer’s disease, blood-brain barrier, neurovascular unit

## Abstract

Cerebral small vessel disease (SVD) refers to a group of disease conditions affecting the cerebral small vessels, which include the small arteries, arterioles, capillaries, and postcapillary venules in the brain. SVD is the primary cause of vascular cognitive impairment and gait disturbances in aged people. There are several types of SVD, though arteriolosclerosis, which is mainly associated with hypertension, aging, and diabetes mellitus, and cerebral amyloid angiopathy (CAA) comprise most SVD cases. The pathology of arteriolosclerosis-induced SVD is characterized by fibrinoid necrosis and lipohyalinosis, while CAA-associated SVD is characterized by progressive deposition of amyloid beta (Aβ) protein in the cerebral vessels. Brain magnetic resonance imaging (MRI) has been used for examination of SVD lesions; typical lesions are characterized by white matter hyperintensity, lacunar infarcts, enlargement of perivascular spaces (EPVS), microbleeds, cortical superficial siderosis (cSS), and cortical microinfarcts. The microvascular changes that occur in the small vessels are difficult to identify clearly; however, these consequent image findings can represent the SVD. There are two main strategies for prevention and treatment of SVD, i.e., pharmacotherapy and lifestyle modification. In this review, we discuss clinical features of SVD, experimental models replicating SVD, and treatments to further understand the pathological and clinical features of SVD.

## Introduction

Advances in neuroimaging technology have led to the recognition of the existence of various small vessel pathologies affecting the brain, including both ischemia and hemorrhages, called cerebral small vessel disease (SVD). The term SVD is an umbrella term used to describe several conditions that share common pathological, clinical, and neuroimaging features (Pantoni, 2010), and the two major types of SVD are arteriolosclerosis, which is mainly associated with hypertension, and cerebral amyloid angiopathy (CAA; Pantoni, 2010; Tomimoto, 2011). Brain magnetic resonance imaging (MRI) shows diverse vascular lesions in patients with SVD, such as white matter lesions, lacunar infarcts, hematomas, microbleeds (MBs), and cortical superficial siderosis (cSS; Pantoni, 2010; Wardlaw et al., 2019). Because MRI technology has allowed for improved visualization of SVD, not only can acute ischemic and hemorrhagic strokes be detected, but also chronic microvascular changes that lead to dementia, as SVD is not only associated with strokes but also with Alzheimer’s disease (Tomimoto, 2011).

Since the underlying pathophysiological mechanisms of SVD are complicated, both *in vivo* and *in vitro* experiments are necessary to understand the disease etiology and the associated vascular changes induced by disease progression. Indeed, clinical features of SVD exhibit heterogeneity ranging from pure vascular disease to admixture with Alzheimer’s disease, and thereby treatment strategy is now controversial. Moreover, recent MRI studies have shown that SVD lesions are detectable in clinical situations, allowing for early identification and treatment of patients with SVD.

Although SVD is associated with pathological and/or functional abnormalities of cerebral small vessels, as its name suggests, SVD shares many characteristics with atherosclerotic large vessel and cardiovascular diseases. Indeed, intracranial carotid arterial calcifications and internal carotid artery stenosis can be associated with changes in the imaging markers used to identify SVD (Chen et al., 2019; Shen et al., 2020), and lobar MBs are also seen in patients with atrial fibrillation (Horstmann et al., 2015). These findings suggest that upstream blood vessels can affect those downstream; this phenomenon is known as “large and small artery cross-talk.”

In this mini review article, we will summarize the experimental models and clinical findings of SVD to further understand the pathological conditions in SVD.

## Types of Cerebral Small Vessel Disease

SVD is the main cause of vascular cognitive impairment, mood disorders, and gait disturbances in aged people (Pantoni, 2010). Most SVD cases are related to hypertension, or other vascular risk factors, and CAA. Although few in number, some patients may develop SVD due to genetic variants that place them at higher risk, such as cerebral autosomal dominant arteriopathy with subcortical infarcts and leukoencephalopathy (CADASIL; Wardlaw et al., 2019) Neuroradiological findings of SVD vary depending on disease etiology. Representative images of SVD typically show white matter hyperintensities (WMH) in MRI, as well as lacunar infarcts, and cerebral MBs (Pantoni, 2010). Moreover, enlargement of perivascular spaces (EPVS), cSS, convexity subarachnoid hemorrhages, and cortical microinfarcts (CMIs) are also indicators of SVD (van Veluw et al., 2017; Wilson et al., 2017; Raposo et al., 2018; Ii et al., 2013, 2019; Wardlaw et al., 2019).

**Figure 1:**
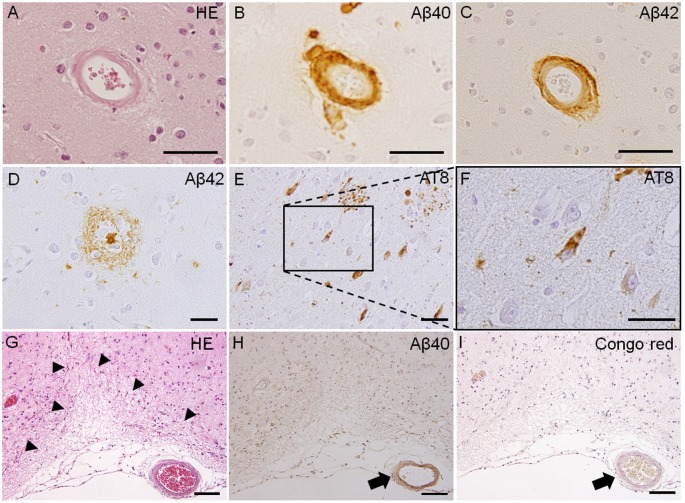
Pathological features of Type 1 and Type 2 cerebral small vessel disease (SVD). Fibrinoid necrosis detected by hematoxylin & eosin (H&E) staining in the postmortem brain of a patient with arteriolosclerosis **(A)**. Immunohistochemistry of the postmortem brain affected by cerebral amyloid angiopathy (CAA) shows immunopositive-staining for beta-amyloid peptides Aβ_1–40_ (Aβ40) and Aβ_1–42_ (Aβ42) in arterioles **(B,C)**. Immunoreactive staining of senile plaque (Aβ40) and neurofibrillary tangles (phosphorylated tau; AT8) is also detected in CAA patients **(D–F)**. Cortical microinfarct stained with HE is observed in a CAA patient (arrowheads, **G**). Aβ40 and Congo red-positive vessels are observed close to the cortical microinfarct (arrows, **H,I**). Scale bars in **(A–F)**: 50 μm; in **(G–I)**: 200 μm.

Because SVD can be caused by a number of different conditions, SVD classification was proposed by Pantoni (2010). The two most common types of SVD are arteriolosclerosis (type 1) and CAA (type 2). Type 1 SVD, caused by arteriolosclerosis, is related to aging and certain vascular factors, such as diabetes and, especially, hypertension (Furuta et al., 1991; Pantoni, 2010). The pathophysiology of type 1 SVD is characterized by fibrinoid necrosis, lipohyalinosis, fibrohyalinosis, microatheroma, and microaneurysm (Pantoni, 2010; Ogata et al., 2011). Type 2 SVD, caused by CAA, is characterized by the progressive deposition of amyloid beta (Aβ) protein in the cerebral vessels; the major peptide isoforms of Aβ mainly consisted of Aβ_1–40_ and Aβ_1–42_ (Figure 1; Thal et al., 2008; Pantoni, 2010). The deposition of Aβ in cerebral vessels appears mainly in leptomeningeal and cortical arteries, and capillary Aβ-deposition is not always confirmed (Thal et al., 2008). Positive immunostaining with Congo red dye and thioflavin S is a specific histopathological feature of CAA (Pantoni, 2010). The capillary type of CAA is associated with advanced stages of Alzheimer’s disease-related pathology, the presence of the apolipoprotein E (APOE) ε4 allele, and complement immune system activation (Matsuo et al., 2018). Moreover, lobar MBs and CMIs are often observed in severe CAA cases, and these lesions are typically associated with cognitive impairments (van Veluw et al., 2016). Other types of SVD include inherited or genetic SVD (Type 3), inflammatory and immunologically mediated SVD (Type 4), SVD caused by venous collagenosis (Type 5), and SVD related to other causes (Type 6; Ii et al., 2019). CADASIL and Fabry disease are well-known causes of inherited or genetic SVD. Inflammatory and immunologically mediated SVD is usually a component of a systemic disease, including Wegener’s granulomatosis, Churg-Strauss syndrome, and microscopic polyangiitis (Pantoni, 2010; Jennette and Falk, 1997). Type 5 SVD, caused by venous collagenosis, is observed in people of advanced age, and the pathological features include noninflammatory collagenosis of venous walls resulting in small vessel narrowing (Moody et al., 1995). Other types of SVD include postradiation angiopathy, as well as nonamyloid microvessel degeneration observed in Alzheimer’s disease (Pantoni, 2010).

## Experiment Models for Evaluation of Small Vessel Disease

Several rodent models have been used to evaluate subcortical white matter changes caused by ischemia. Although animal models of chronic cerebral hypoperfusion cannot explain all features of SVD in humans, large vessel occlusion/stenosis models are widely accepted animal models of SVD for assessing large and small artery cross-talk. Two-vessel occlusion of the common carotid arteries is a common rat model of white matter ischemia (Wakita et al., 1994) in which a permanent occlusion causes pathophysiological changes in the corpus callosum, the internal capsule, and the optic nerve (Wakita et al., 2002; Farkas et al., 2004), and white matter demyelination similar to the white matter lesions in humans. Although this model results in analogous neuropathological changes, the visual pathway is damaged by the carotid artery occlusion, making it difficult to assess neuropsychological changes in behavioral testing paradigms (Farkas et al., 2004). To counteract these drawbacks, we have developed mice models which are subjected to bilateral carotid artery stenosis (BCAS) by attaching microcoils from outside of both carotid arteries (Shibata et al., 2004, 2007). This model exhibits white matter lesions and cognitive decline after chronic cerebral hypoperfusion for 1 month, and delayed hippocampal atrophy at 8 months after chronic cerebral hypoperfusion (Nishio et al., 2010), whereas there are little optic nerve damages and visual impairment. This BCAS mouse model has been most widely accepted as a model for vascular dementia with white matter pathologies, such as demyelination, axonal damage, oligodendrocyte loss, and blood-brain barrier (BBB) damage (Miyamoto et al., 2014). Because the two-vessel occlusion/stenosis model acutely decreases cerebral blood flow (CBF), a device that can narrow the arteries gradually has also been introduced. Ameroid constrictor devices applied to the bilateral common carotid arteries could replicate white matter pathologies, can reduce CBF gradually, and can induce changes resembling those associated with chronic cerebral hypoperfusion in humans (Kitamura et al., 2012; Hattori et al., 2016). The gradual common carotid artery stenosis (GCAS) model gradually and continuously reduces CBF (Hattori et al., 2016). A mouse asymmetric common carotid artery stenosis (ACAS) model is used to mimic white matter infarcts accompanied by motor deficits and dementia; the model is generated by the implantation of an ameroid constrictor and a microcoil in both carotid arteries (Hattori et al., 2015). The spontaneously hypertensive rat two-vessel gradual occlusion (SHR-2VGO) model is also generated using ameroid constrictors, and is used to model the evolution of white matter abnormalities and the associated impairments of spatial working memory (Kitamura et al., 2015). A nongenetic model of SVD is the stroke-prone spontaneously hypertensive rat (SHRSP), in which this rat strain has malignant hypertension, and reveals white matter loss and reductions in the levels of tight junction proteins (Yamori and Horie, 1977; Hainsworth and Markus, 2008; Bailey et al., 2011; Rajani and Williams, 2017).

Focal injection of vasoconstrictors has been used to model certain characteristics of human white matter stroke such as lacunar infarcts. For example, direct stereotaxic injection of vasoconstricting agents such as endothelin-1 (ET-1) and N5-(1-iminoethyl)-L-ornithine (L-NIO) into subcortical white matter can induce focal strokes in both rats and mice (Sozmen et al., 2009, 2012; Hinman et al., 2013). ET-1 can also reduce local blood flow, causing ischemic lesions, and injection into the white matter can induce a demyelinated and necrotic region in rats and a demyelinated area in mice (Silasi et al., 2015). Likewise, L-NIO injection can also cause focal white matter strokes in animals (Hinman et al., 2013).

Transgenic mouse lines for SVD are used to further understand the mechanisms of CAA and CADASIL. Although CAA is often seen sporadically, CAA can also be genetically inherited. Certain mutations in the amyloid precursor protein (APP) gene are a common cause of hereditary CAA. Dutch APP (E693Q) mice accumulate amyloid deposits in brain vessels at ~22–25 months, resulting in brain hemorrhages (Herzig et al., 2004). APP23 transgenic mice, which express mutant human APP_751_, develop Aβ deposits in the neocortex and hippocampus (Sturchler-Pierrat et al., 1997), and develop similar Aβ deposits in cerebral vessels as those caused by CAA beginning at 9 months of age (Kuo et al., 2001). Another transgenic mouse line expressing human Swedish, Dutch, and Iowa triple-mutant APP (Tg-SwDI) has been used to model CAA as well (Davis et al., 2004). Tg-SwDI mice develop microvascular Aβ deposition in the thalamus and subiculum, and exhibit apoptotic vascular cells, and a loss of smooth muscle cells in vessel walls (Miao et al., 2005). Moreover, models combining chronic hypoperfusion models and transgenic mice have been reported, and chronic hypoperfusion could exacerbate BBB dysfunction in APP23 transgenic mice and increase the frequency of microinfarctions (Salvadores et al., 2017; Shang et al., 2019). On the other hand, CADASIL is linked to a mutation in the *NOTCH3* gene, with the pathological feature being the accumulation of granular osmiophilic material within the tunica media of vascular smooth muscle cell membranes (Ayata, 2010). *Notch3* knockout, knock-in, and transgenic mouse models, and *notch3* mutant zebrafish have been developed to better understand the pathological features of CADASIL (Ayata, 2010; Zaucker et al., 2013; Cognat et al., 2014). These models have demonstrated that white matter deficits are associated with oligodendrocyte death (Cognat et al., 2014), reduced expression of myelin basic protein, and a decreased number of oligodendrocyte progenitor cells (OPCs; Zaucker et al., 2013).

Several kinds of *in vitro* models are used for the evaluation of cellular changes associated with SVD, such as oxygen-glucose deprivation, hypoxia, growth factor deprivation, and glutathione depletion (Shindo et al., 2016). To mimic chronic mild hypoxic conditions, cobalt chloride was selected for use in some of these experiments (Miyamoto et al., 2013; Shimada et al., 2019). Recently, induced pluripotent stem (iPS) cell technology (Takahashi and Yamanaka, 2006) has been acknowledged as a useful cellular tool for the evaluation and treatment of various disease conditions, and iPS cell derived from the dermal fibroblasts of the patients with hereditary cerebral hemorrhage with amyloidosis-Dutch type (HCHWA-D) is considered to be a model for sporadic CAA (Daoutsali et al., 2019). Moreover, CADASIL and Fabry disease iPS cells have been used in several studies (Kawagoe et al., 2013; Ling et al., 2019). It is desired that these iPS cell-based SVD models can be used to help understand the pathogenic mechanisms of SVD and lead to the development of novel treatment strategies.

## Neuroimaging of Cerebral Small Vessel Disease (Figure 2)

Brain MRI is typically used to examine SVD lesions, which are often characterized by WMH, lacunar infarcts, EPVS, MBs, cSS, and CMIs (Wardlaw et al., 2019).

**Figure 2:**
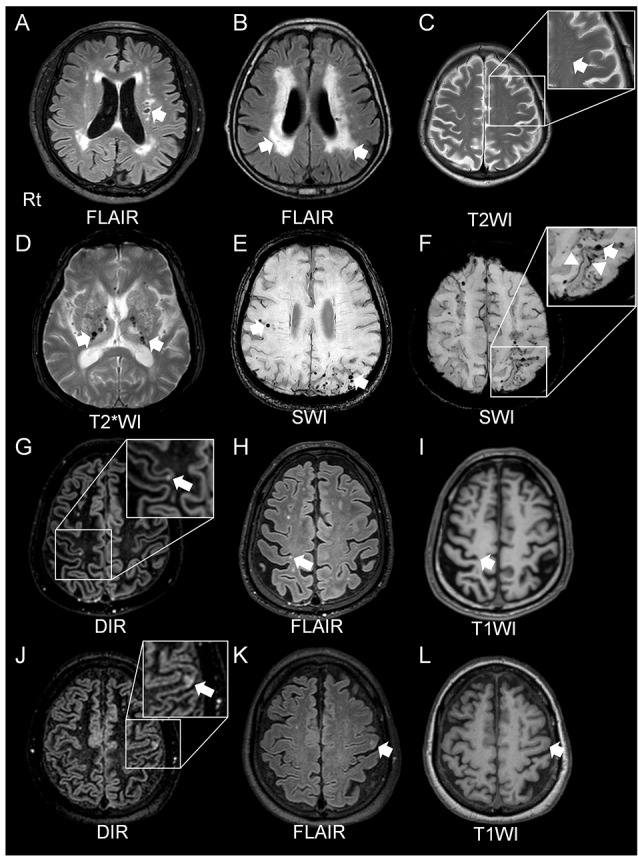
Radiological features of cerebral SVD. A lacunar infarct in the left corona radiata **(A)** and white matter lesions, assessed by fluid attenuated inversion recovery (FLAIR) imaging, is detected in patients with hypertension (arrows, **A,B**). The arrow shows enlarged perivascular spaces in T2-weighted (T2WI) magnetic resonance imaging (MRI) in a patient with CAA **(C)**. Cerebral microbleeds (MBs) are classified as two types: deep MBs or lobar MBs. Deep MBs are detected in patients with hypertension on MRI T2*-weighted image (T2*WI; arrows, **D**). Both lobar MBs and cortical superficial siderosis (cSS) are seen in susceptibility-weighted imaging (SWI) sequences in CAA patients (arrows, **E,F**, and arrowheads, **F**). Both double inversion recovery (DIR; **G,J**) and FLAIR **(H,K)** imaging can clearly detect the cortical microinfarcts (CMIs). T1*-weighted imaging (T1WI) can also reveal CMIs but less obviously **(I,L)**. CAA and cerebral embolisms can cause CMIs. CMIs from patients with CAAs **(G–I)** showed that all lesions were localized within cortical structures, with a size of <5 mm (Ishikawa et al., 2020). CMIs caused by embolisms involved subcortical areas, and the size of the lesions was ≥5 mm (Ishikawa et al., 2020).

WMH are often observed in both stroke patients and in nonstroke elderly people (Longstreth et al., 2002; Vermeer et al., 2002). In MRI, these hyperintensities are observed on T2-weighted images (WI) and fluid-attenuated inversion recovery (FLAIR), while hypointensities are observed on T1-WI, with no low intensity cavities in FLAIR images (Wardlaw et al., 2019). Although both hypertensive SVD and CAA show WMH in MRI on T2-WI and hypointensities on T1-WI, the distribution of these hyperintense regions differs; peri-basal ganglia WMH are strongly indicative of hypertensive arteriopathy, while multiple punctate FLAIR images are associated with CAA (Charidimou et al., 2016). Lacunar infarcts are round or ovoid, subcortical, fluid-filled cavities, 3–15 mm in diameter (Wardlaw et al., 2013). A common imaging feature of lacunar infarcts is a central hypointensity with a hyperintense rim in FLAIR images, but there are some cases without a central hypointensity lesion (Wardlaw et al., 2013). These WMH and silent lacunar infarcts can become important stroke risk factors, and might be associated with increased mortality rates (Bokura et al., 2006). EPVS are important markers of vascular dysfunction leading to brain damage (Wardlaw et al., 2019). MRI findings of EPVS are similar to cerebrospinal fluid on all sequences; they can be linear, round, or ovoid lesions with a diameter generally smaller than 3 mm (Wardlaw et al., 2013). The perivascular space is considered an important feature of glymphatic pathways as that is one of the main clearance pathways for the removal of amyloid-beta peptides, and changes in these areas are indicated in increased risk of Alzheimer’s disease (Iliff et al., 2015).

Cerebral MBs are detected as small, hypointense, round lesions with a diameter of 5–10 mm on T2*-weighted gradient-echo (GRE) or susceptibility-weighted image (SWI) sequences (Fazekas et al., 1999; Charidimou et al., 2013). Two main types contribute to pathological features of cerebral MBs have been identified: hypertensive vasculopathy and CAA (Greenberg et al., 2009). Differential distribution patterns of these two types of MBs are observed; hypertensive vasculopathy is typically associated with deep MBs in the basal ganglia, thalamus, and brainstem, while advanced CAA is associated with lobar MBs (Greenberg et al., 2009). Moreover, there is a possibility of mixed types of MBs, which may indicate advanced hypertensive arteriopathy, or alternatively both hypertensive arteriopathy and CAA (Matsuyama et al., 2017; Pasi et al., 2018). On the other hand, based on the pathophysiological mechanisms producing these MBs, they can be classified as either primary or secondary (Fisher, 2014). Primary cerebral MBs are visualized as extravasated erythrocytes from cerebral small vessels upon histopathological examination (Fazekas et al., 1999); secondary MBs are caused by other mechanisms such as hemorrhagic transformation of cerebral infarction/microinfarction (Fisher, 2014; Ito et al., 2019; Ogawa Ito et al., 2019).

The MRI finding of cSS is a linear or curvilinear lesions at the gyral cortical surface, detected as a signal loss on T2*-weighted GRE and SWI sequences (Charidimou et al., 2015a). cSS is one of the key features of CAA and is associated with transient focal neurological episodes (Charidimou et al., 2012, 2015a). Moreover, cSS is a risk factor for intracerebral hemorrhage in CAA patients (Charidimou et al., 2015b), and multifocal cSS correlates with disease severity in patients with CAA (Charidimou et al., 2017). Although the neuropathological feature of cSS may be associated with complement immune system activation (Matsuo et al., 2018), the details of this possible relationship remain unclear.

CMIs are frequently detected in autopsied brains of elderly people. Even though there is no visible macroscopic cerebral infarct, CMIs are frequently associated with cognitive impairment (Brundel et al., 2012; Kövari et al., 2017). CMIs are caused by various pathologies such as CAA, arteriolosclerosis, and microembolisms (Kövari et al., 2017). Although detection of CMIs has been difficult, historically, using MRI (Smith et al., 2012), recent reports have shown that *in vivo* visualization of MCIs is possible using higher resolution 7-Tesla (7T; van Veluw et al., 2013) and 3-Tesla (3T; Ii et al., 2013; van Veluw et al., 2015) MRI, and a combination method using three-dimensional double inversion recovery (3D-DIR) and three-dimensional FLAIR (3D-FLAIR) imaging with 3T-MRI has reportedly been able to detect CMIs (Ii et al., 2013; Umino et al., 2019). MRI features of CMIs show small, high-intensity intracortical lesions on 3D-DIR and 3D-FLAIR imaging, and hypointensities on T1-WI. Histopathological studies have demonstrated neuroradiological and pathological correlations (Niwa et al., 2017; Ishikawa et al., 2018). A recent study using 3T-MRI has shown that it may be possible to distinguish between CMIs due to CAA and those due to microembolism (Ishikawa et al., 2020). They say that CMIs in CAA patients are localized within the cortex, predominantly in the occipital lobe, smaller <5 mm in diameter, with fewer than three lesions (Ishikawa et al., 2020). On the other hand, CMIs resulting from microembolisms may involve the cortico-subcortical junction, are predominantly distributed in the frontal or parietal lobes, larger (≥5 mm) in diameter with multiple lesions, typically at least three (Ishikawa et al., 2020).

## Clinical Symptoms and Treatment of Cerebral Small Vessel Disease

Symptoms of SVD vary depending on several factors, including the type and localization of vascular lesions. For lacunar infarcts, the symptoms include acute lacunar motor and/or sensory syndromes. Total number and volume of SVD lesions can be associated with cognitive impairment, gait disturbance, and mood disorders (Wardlaw et al., 2019). Even though MRI may show several SVD-related lesions, some patients may have symptoms that are clinically silent or may even be asymptomatic (Pantoni, 2010; Huijts et al., 2013). To determine the total MRI burden of SVD, calculation of a total SVD score has been described (Klarenbeek et al., 2013), based on four MRI features of SVD, including the presence of lacunae, the presence of MBs, the severity of the basal ganglia perivascular space, and the degree of WMH. This weighted score has been shown to be associated with age, sex, and vascular risk factors, including hypertension and smoking (Staals et al., 2014). Moreover, the total SVD score has been associated with cognitive impairment (Huijts et al., 2013), and may have predictive value for assessing risk of recurrent stroke after ischemic stroke (Lau et al., 2017), as well as mild parkinsonian signs (Hatate et al., 2016).

There are two main approaches of prevention and treatment strategies for SVD: pharmacotherapy and lifestyle modification. Pharmacotherapy includes anti-hypertensive drugs, statins, and antiplatelet treatments. Because hypertension is a risk factor for SVD, one of the most important treatments is anti-hypertensive therapy. The secondary prevention of small subcortical stroke (SPS3) trial enrolled patients with symptomatic lacunar infarctions, which were divided into two groups: those with a systolic blood pressure target of 130–149 mm Hg and those with a blood pressure less than 130 mm Hg (Benavente et al., 2013). Although there was no significant difference in the frequency of all strokes, the rate of intracerebral hemorrhage was significantly lower in the group with lower blood pressure (Benavente et al., 2013). This SPS3 trial showed that a systolic blood pressure target of less than 130 mm Hg is beneficial in reducing risk in lacunar stroke patients. Moreover, a systematic review of SVD treatment showed that anti-hypertensive treatment has a protective role in limiting the progression of WMH, but does not affect the degree of brain atrophy (van Middelaar et al., 2018). Statins may have beneficial effects for SVD patients, as rosuvastatin has been shown to prevent WMH in patients expressing the APOEε4 allele (Ji et al., 2018). Diabetes mellitus is also associated with SVD, especially lacunar stroke and the low fractional anisotropy observed on MRI (Liu et al., 2018). Most patients with diabetes have another risk factor, such as hypertension, and blood pressure and lipid control is effective for stroke prevention (Chen et al., 2016).

In terms of antiplatelet treatment, the SPS3 trial also demonstrated that all-cause mortality was increased in dual antiplatelet therapy with aspirin and clopidogrel, and that there was no significant difference in recurrent stroke risk between single antiplatelet therapy and dual antiplatelet therapy in lacunar stroke patients (Benavente et al., 2012). A recent meta-analysis showed a significant risk reduction in recurrence of any type of stroke and ischemic stroke in patients with a lacunar stroke using any single antiplatelet medication (Kwok et al., 2015). Likewise, cilostazol, a phosphodiesterase 3 inhibitor, may have an advantage for treating lacunar stroke. A randomized double-blind, placebo-controlled trial in Japan, the Cilostazol Stroke Prevention Study (CSPS), showed that cilostazol reduced the risk of recurrent stroke compared with placebo (Matsumoto, 2005), and the Cilostazol for Prevention of Secondary Stroke study (CSPS2) demonstrated that there was no significant difference in stroke prevention measures between cilostazol and aspirin, but cilostazol resulted in fewer hemorrhagic events than aspirin alone (Shinohara et al., 2010). Moreover, dual antiplatelet therapy with cilostazol and aspirin or clopidogrel can reduce the incidence of ischemic stroke recurrence and bleeding compared with aspirin or clopidogrel alone (Toyoda et al., 2019). In addition, *in vivo* experiment data has shown that cilostazol had a protective effect against hypertension-induced endothelial dysfunction (Oyama et al., 2011), and may reduce the risk of developing dementia (Tai et al., 2017; Saito et al., 2019). These data may suggest that cilostazol can be a safe and effective treatment for SVD in certain cases.

Lifestyle modification is another important intervention for preventing and treating SVD. Smoking and high sodium diets exacerbate the risk of stroke, dementia, and WMH revealed in MRI (Staals et al., 2014; Karama et al., 2015; Hankey, 2017; Wardlaw et al., 2019). The Finnish Geriatric Intervention Study to Prevent Cognitive Impairment and Disability (FINGER) trial demonstrated that multidomain intervention, including diet, exercise, cognitive training, and vascular risk monitoring, improved cognitive function and reduced the risk of cognitive decline in elderly subjects (Ngandu et al., 2015). Although the number of patients enrolled was relatively small, another study showed that an aerobic exercise training program might have beneficial effects for patients with subcortical ischemic vascular cognitive impairments (Liu-Ambrose et al., 2016).

## Conclusion

SVD is clinically important because it is not only associated with ischemic or hemorrhagic stroke but also with dementia. As SVD is an umbrella term for various conditions affecting cerebral vasculature, clinical characteristics of SVD can vary greatly depending on the etiology, which can affect optimization of treatment. In this mini review article, we have summarized the experimental models and the pathological and radiological features of SVD. Further elucidating the characteristics and pathophysiology of SVD may help identify novel therapeutic approaches and allow for earlier diagnoses to protect the human brain from disease progression.

## Ethics Statement

The study was approved by the Ethics Committee of Mie University Hospital (number H2018-032).

## Author Contributions

AS: draft of manuscript, review concept and design, and acquisition of data. HI, YI, and AN: revision of manuscript and acquisition of data. HT: revision of the manuscript and review supervision.

## Conflict of Interest

The authors declare that the research was conducted in the absence of any commercial or financial relationships that could be construed as a potential conflict of interest.
